# Maternal, fetal and neonatal outcomes among pregnant women with arthrogryposis multiplex congenita: a scoping review

**DOI:** 10.1186/s13023-025-03631-5

**Published:** 2025-03-17

**Authors:** Arda Arduç, Johanna I. P. De Vries, Maria B. Tan-Sindhunata, Femke Stoelinga, Remco Jansen, Ingeborg H. Linskens

**Affiliations:** 1 Department of Obstetrics and Gynecology, Amsterdam UMC, University of Amsterdam, Meibergdreef 9, 1105 AZ Amsterdam, The Netherlands; 2 Amsterdam Reproduction and Development, Amsterdam, The Netherlands; 3 Department of Human Genetics, Amsterdam UMC, University of Amsterdam, Amsterdam, The Netherlands; 4 Department of Rehabilitation Medicine, Amsterdam UMC, University of Amsterdam, Amsterdam, The Netherlands; 5 Spierziekten Nederland, Patient Support Group, Focusgroup Arthrogyposis Multiplex Congenita, Baarn, The Netherlands

**Keywords:** Arthrogryposis multiplex congenita, Pregnancy outcome, Mode of delivery, Pregnancy complications, Preterm birth, Small for gestational age

## Abstract

**Background:**

The rarity of pregnancies in women with arthrogryposis multiplex congenita (AMC) could lead to healthcare providers having limited exposure to these cases. Consequently, they may be less familiar with the possibilities and challenges associated with pregnancies in women affected by AMC. AMC is an umbrella term for a disorder with multiple contractures at birth, having a broad spectrum of causes, onset and severity of expression. A clinical classification describing the phenotype is Group 1 with primary limb involvement, Group 2 with musculoskeletal involvement plus other system anomalies, and Group 3 with musculoskeletal involvement plus central nervous system dysfunction and/or intellectual disability. A scoping review was conducted to review available literature on documented cases of pregnancies in women with AMC, with the following aims: (1) to outline the maternal, fetal and neonatal outcomes; (2) to describe AMC stability during and after pregnancy (worsening of symptoms due to contractures, increased muscle weakness, pain or lung involvement); and (3) to summarize counselling aspects during pregnancy for expecting mothers who have AMC.

**Results:**

This scoping review included 27 manuscripts reporting on 43 women with 82 pregnancies, of whom 18 in Group 1, 20 in Group 2, 2 in Group 3, and 3 with an unknown type. Details on pregnancy-related outcomes could be depicted from 26 of the 43 women concerning 31 pregnancies. Among these pregnancies, 74% (23/31) had a cesarean section delivery, of which 74% (17/23) were elective. Children were born preterm before week 37 in 7 of 31 pregnancies (22%). A birth weight below the 10th percentile was seen in 6 of the 24 (25%) with a reported birth weight. The course of the pregnancy was uneventful in 16 of the 26 women (62%). Pregnancy had a limited negative influence on AMC stability except for three cases with a transient worsening of lung function.

**Conclusions:**

Gathering the information of the case histories revealed that the majority of the reported women had Distal Arthrogryposis with stable AMC during pregnancy and after delivery. The risk to have a cesarean section, preterm labour or a small for gestational age child is higher in this group than in the general population. Insights obtained by this review emphasized to offer (pre)pregnancy counselling and care by a multidisciplinary team tailored to the women’s type of AMC, to ensure optimal preparation for both obstetric, genetic, neurologic, pulmonary and anesthetic care during pregnancy, delivery and postpartum period.

**Supplementary Information:**

The online version contains supplementary material available at 10.1186/s13023-025-03631-5.

## Background

Arthrogryposis multiplex congenita (AMC) is a group of rare diseases occurring in 1 in 3000–5200 live births [1, 2]. Healthcare providers have therefore a limited exposure to the possibilities and challenges associated with pregnancies in women affected by AMC. AMC is phenotypically characterized by multiple joint contractures manifesting in diverse anatomical regions and varying degrees of severity [1]. Its etiology is varied and includes genetic and non-genetic factors, including neuromuscular conditions, maternal illnesses, and limited intrauterine space [1–3]. The type of AMC was grouped into three groups depending on involvement according to Hall et al. [1]: Group 1 with primary limb involvement, Group 2 with musculoskeletal involvement plus other system anomalies, and Group 3 with musculoskeletal involvement plus central nervous system dysfunction and/or intellectual disability.

Several evaluations in adults with AMC revealed a higher quality of life compared to the general population, despite the prevalent experiences of pain and fatigue in individuals with AMC [4–13] Notably, half of the adults with AMC lead independent lives with active engagement in work and social spheres, with the other half requiring some level of assistance [5, 9]. Given their productive lives, it is understandable that pregnancy-related questions such as child wish arise among women with AMC [4, 14, 15]. A previous study showed a higher risk of adverse outcomes in pregnant women with physical (e.g. cerebral palsy), intellectual (e.g. DiGeorge syndrome), and sensory conditions (e.g. glaucoma) [16]. Accessibility to maternity care remains limited for these women [17]. Women with physical disabilities experience various challenges, including physical barriers, communication knowledge deficits with healthcare providers and limited accessibility to maternity care such as wheelchair accessible rooms and equipment adapted to their needs [17]. Additionally, a survey among women with AMC has also emphasized the need for information on pregnancy-related topics [4].

Prompted by international patient support groups for AMC, a scoping review was conducted to evaluate whether pregnant women with AMC are at risk of complications for themselves, their fetus, or newborns to address the knowledge gap regarding pregnancy outcomes in women with AMC. Our focus was on maternal, fetal and neonatal outcomes, including maternal stability of AMC during and after pregnancy (worsening of symptoms due to the contractures, increased muscle weakness, pain or lung involvement), and counselling for women before and during their pregnancies. The insights gained by this literature review will increase awareness among healthcare providers and women with AMC about the possibilities and challenges during pregnancy.

## Methods

A scoping review was conducted to better understand what is known about pregnancies among women with AMC [18–20]. Specifically, we addressed the following aims: (1) outline the maternal, fetal and neonatal outcomes; (2) describe AMC stability during and after pregnancy (worsening of symptoms due to the contractures, for example increased muscle weakness, pain or lung involvement); and (3) summarize counselling aspects during pregnancy for expecting mothers who have AMC.

**Fig. 1:**
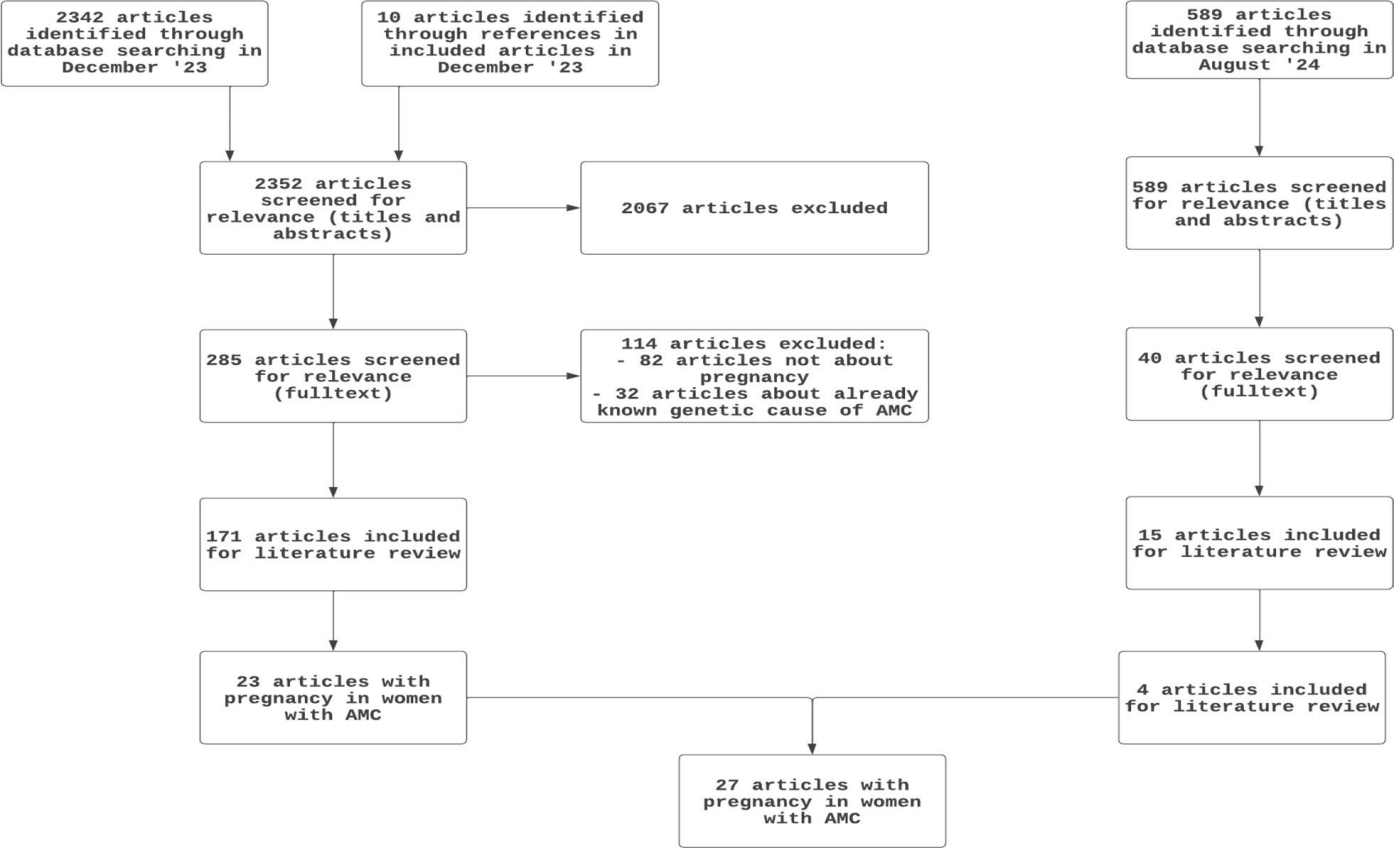
Flowchart of the literature search on pregnancies in women with AMC by using the PRISMA criteria

A systematic search of the literature was performed in the following databases: PubMed, Embase, and Web of Science. The timeframe within the databases was from inception to 5th August 2024 and conducted by the librarians. The search included keywords and free text terms for (synonyms of) 'arthrogryposis' combined with (synonyms of) 'pregnancy' combined with (synonyms of) 'data collection method'. Selection of manuscripts is done by applying all manuscripts related to maternal, fetal, and neonatal outcomes in pregnancy in women with AMC or counselling aspects (Fig. 1). A full overview of the search terms per database can be found in the supplementary information (Additional File 1). No limitations on date or language were applied in the search. The PRISMA-ScR checklist for scoping reviews was used to guide the conduct of this review (Additional File 2) [20].

The Rayyan website (www.rayyab.qcri.org), which supports storage, multi-person selection, and grouping of the manuscripts, was used for the selection of manuscripts following a de-duplication process. Two investigators (AA and JIPdV) completed the screening process at the titles and abstracts, and full texts independently in a blinded fashion. Manuscripts that were not selected by both investigators were excluded for this review and a third reviewer was not consulted in case of disagreements. The residual manuscripts were assessed again on relevance by both investigators together. The quality of the included studies was not appraised. Data extraction was done by the two investigators.

The type of AMC was grouped into three groups depending on clinical involvement according to Hall et al [1, 21]: Group 1 with primary limb involvement, Group 2 with musculoskeletal involvement plus other system anomalies, and Group 3 with musculoskeletal involvement plus central nervous system dysfunction and/or intellectual disability.

The information in these manuscripts was finally categorized in the following aspects:

1. Maternal, fetal and neonatal outcomes.
2. AMC stability during and after pregnancy: maternal difficulties were extracted from the included studies, such as worsening of symptoms due to the contractures, increased muscle weakness, pain, or lung involvement.
3. Counselling aspects in AMC: corresponding advice provided in the various manuscripts were gathered.

## Results

The search yielded a total of 211 manuscripts about AMC and pregnancy; 27 met the inclusion criteria and were included in this review (Fig. 1). These 27 reports included 43 women and 82 pregnancies (Table 1) [22–46].

**Table 1:** Maternal characteristics. Classification based on involvement of AMC with primary extremities (Group 1), with musculoskeletal and other system anomalies (Group 2), and with musculoskeletal plus central nervous system dysfunction and/or intellectual disability (Group 3) according to Hall et al. [1]. UK = unknown

Case	Author	Type of AMC AMC group (hall’s classification) diagnosis clinical/genetic diagnosis	Inheritance autosomal dominant (AD), autosomal recessive (AR),- (not inheritable), UK (unknown)	Body parts involved by AMC and mobility walking, wheelchair bound, imobilty	Age during pregnancy (years)
1	Moore et al., 1989	2, clinical	AD	Upper and lower limbs, fingers, hip and feet, craniofacial abnormalities. Walking	21
2	Moore et al., 1989	2, clinical Mother of case 1	AD	Upper and lower limbs, wrist, knees, foot, ankle and toes, craniofacial abnormalities. Walking	20
3	Hackett et al., 2000	1 (Amyoplasia), clinical	-	Upper and lower limbs, shoulders, elbows, wrists, fingers, hips, knees, feet and spine, scoliosis. Walking	22
4	Baty et al., 1988	1, DA (Type I), clinical	AD	Upper and lower limbs, wrist (limited), foot, ankle and toes. Walking	A.26 B. 29
5	Quance et al., 1988	3*, clinical	UK	Upper and lower limbs, spine (scoliosis), neck, pelvis, Facial and neuromuscular abnormalities. Wheelchair bound	21
6	Rozkowski et al., 1996	3*, clinical	UK	Upper and lower limb, pelvis, spine (kyphoscoliosis), neuromuscular abnormalitie.s Wheelchair bound	34
7	Gripp et al., 1996	1, clinical	AD	Upper and lower limbs, hips, knees and feet Walking, stiff gait	23
8	Spooner et al., 2000	2*, clinical	UK	Upper and lower limbs, hips, spine (kyphoscoliosis), narrow pelvis. Walking unaided, but use a wheelchair	26
9	Hardwick et al., 2002	2*, clinical	AR	Upper and lower limbs, hips and elbow, length 1.21 m Spine: limited neck extension and severe kyphoscoliosis. Walking with stich “swing-through gait”, no mobility in the legs	27
10	Leeners et al., 2005	2 (Freeman-Sheldon syndrome), Clinical	AD	Feet. Walking	22
11	Duffy et al., 2007	2*, clinical	UK	Upper and lower limbs, spine severe thoracic kyphosis and lumbar lordosis, normal neck extension, Stature 0.91m, BMI 31. Walking (although little mobility in the legs)	27
12	Singhal et al., 2010	2*, clinical	UK	Upper and lower limbs, hands and feet, spine: no involvement neck, thoracic kyphosis and lumbar lordosis, short stature 1.3m. No walking, crawling	25
13	Ko et al., 2013	2, Sheldon-Hall syndrome, Genetic: Heterozygous TPM2 mutation p.R133, autosomal dominant	AD	Upper and lower extremities, hands and feet, camptodactyly, short stature 1,53m, short neck sloping shoulders, facial anomalies, retrognathia. Independent walking	25
14	Castro et al., 2013	2,*, clinical	UK	Lower limbs, hips and spine. Lumbar scoliosis, pelvis, short stature 1.2m. Independent walking with crutches	36
15	Darwich et al., 2014	2*, clinical	UK	Limbs, spine severe kyphoscoliosis, restrictive lung disease, laryngeal surgery for vocal cord dysfunction, short stature 1,5m, weigh 30.8, BMI 13. Class 1 airway, small thyromental distance, stiff temporomandibular joint. Walking	26
16	Sadacharam et al., 2016	2*, clinical Diabetes, gastroesophageal reflux	UK	Upper and lower limbs and spine, severe thoracolumbar kyphoscoliosis. Reduced left lung volume, Class I airway, normal neck extension Walking	28
17	Guzman-Lopez et al., 2019	1*, clinical	UK	Upper and lower limbs, hips, coxofemoral prothesis. Wheelchair bound	19
18	De Burca et al., 2019	2, DA (type IIB), clinical Heterozygous TNNT3 mutation	AD	Upper and lower limbs, hip. Walking	30
19	Kawira et al., 1985	2, Klippel-Feil, clinical	AD	Upper and lower limbs, scoliosis, axillary pterygia, unusual facial appearance, joined vertebrae, axillary webbing, stature 1,43m, weigh 40kg. Walking	22
20	Pollazzon et al., 2021, Pt 2	1, DA (Type I), clinical and genetic: Variants in the TPM2 c.463G > A, p.(A155T)	AD	Upper and lower limbs. Walking	20
21	Pollazzon et al., 2021, Pt 3, mother of 37	1, DA (Type I), clinical and genetic: Variants in the TPM2 c.463G > A, p.(A155T)	AD	Upper limbs, scoliosis. Walking	UK
22	Pollazzon et al., 2021, Pt 8	2, DA (Type 2B), clinical and genetic: Heterozygous deletion in TNNI2 gene	AD	Upper and lower limbs, micro-, retrognathia. Walking	UK
23	Pollazzon et al., 2021, Pt 12	1 (Amyoplasia), clinical	-	Upper and lower limbs, scoliosis. Walking with orthoses	A. 25 B. UK
24	Llewellyn and Volikas, 2021 (conference abstract)	UK	UK	At least lower limbs + hip deformities. Wheelchair dependent	37
25	Sherlaw-Sturrock et al., 2022	2, DA (Type 5), clinical: heterozygous pathogenic variant in PIEZO2, autosomal dominant	AD	Upper and lower limbs, kyphosis. Progressive restrictive lung disease. Unknown mobility	2 × UK
26	Serra et al., 2022	2, DA (Type 5), clinical: heterozygous pathogenic variant in PIEZO2, autosomal dominant	AD	Upper and lower limbs. Unknown mobility	UK
27	Tang et al., 2020	2, Sheldon Hall Syndrome, clinical	AD	Hands. Walking	UK
28	Tang et al., 2020	2, Sheldon Hall Syndrome, genetic, c.188G > A variant of TNNT3 gene, autosomal dominant	AD	Hands. Walking	UK
29	Tang et al., 2020	2, Sheldon Hall Syndrome), genetic, c.188G > A variant of TNNT3 gene, autosomal dominant	AD	Hands. Walking	UK
30	Böckel et al., 1984	2, clinical	AR	Upper and lower limbs, Multiple pterygium syndrome, short stature, 1,4m. Walking	UK
31	Carlson et al., 1985	UK	UK	UK	UK
32	Carlson et al., 1985	UK	UK	UK	
33	Stoll et al., 1996	1, DA (Type I), clinical	AD	Upper limbs, severe clinodactyly and camptodactyly (grandmother of proband). Walking	UK
34	Stoll et al., 1996	1, DA (Type l), clinical	AD	Clinodactyly and mild camptodactyly of the fifth fingers (mother of proband). Walking	19
35–43	Jiang et al., 2004	1, DA (Type I), clinical	AD	Upper and lower limbs, talipomanus and talipes equinovarus. Unknown mobility	UK

^*^Classification was not provided in the manuscript but could be interpreted from the provided clinical description

### Maternal, fetal and neonatal outcomes

Median maternal age during pregnancy was 25 years (range: 19–37 years). AMC group distribution was 18 women in Group 1, 20 women in Group 2, two in Group 3, and three with an unknown type. Regarding physical ability, at least 26 women (60%) were able to achieve independence in mobility with or without aids. The assumed inheritance (based on the clinical presentation and family history) was known in 29 of the 43 women: autosomal dominant in 27, and autosomal recessive in 2. Genetic causes were found in nine women. Characteristics of the women concerning AMC groups, inheritance, involved body parts and maternal age during gestation are presented in Table 1.

Details on pregnancy-related outcomes could only be depicted from the first 26 women with in total 31 pregnancies (Table 2). Among these pregnancies, 74% (23/31) had a cesarean section delivery, of which 74% (17/23) were elective. The remaining 26% (8/31) of deliveries were vaginal, with an uncomplicated labour in 5 cases, forceps extraction in 1 case, and vacuum extraction in another. Children were born preterm (before week 37) in 7 of 31 pregnancies (22%). A birth weight below the 10th percentile was seen in 6 of the 24 (25%) with a reported birth weight. The course of the pregnancy was uneventful in 16 of the 26 women (62%), without reported pain, premature labour, lung problems, or difficulties during analgesia. Only one of the manuscripts reported on hypertensive disorder of pregnancy. Miscarriage percentage, or fertility issues were not reported in the manuscripts. Maternal and fetal outcomes are presented in Table 2.

**Table 2:** (Pre)pregnancy outcomes of the mothers, fetuses and neonates. US = ultrasound examination, UK = unknown, GA = gestational age

Case	Number of preg-nancies	Multidisciplinary team involved? yes/no/unknown = UK	Genetic testing during pregnancy?	Suspicion of fetus with amc?	AMC/ pregnancy related challenges during pregnancy?	Mode of delivery vaginal, emergency or elective cesarean section (EM SC/ EL SC)	Gestational age in weeks	AMC/ pregnancy related difficulties during delivery? yes/no, Including type of anesthesia
1	1	UK	No	No: serial US, GA unknown	No fetal movements till 24th week	Vaginal, forceps extraction for fetal distress	Term	No
2	3	UK	No	No 3x	No	Vaginal 3x	term 3x	No
3	1	Yes (occupational therapist, social worker, neonatologist, nurses, obstetricians, anesthetist)	No	No: serial US (no contractures, normal limb and body movements), GA unknown	Immobility due to pain and maternal discomforts	Induced, maternal indication, vaginal, vacuum extraction for failure of progress	37	No
4	2	UK	No	A. No B. Yes, US at 19 and 22 wks: wrist and finger contractures, 28 wks also feet	A. spontaneous premature rupture of membranes B. No	A. EM SC for fetal distress B. EL SC for cephalopelvine disproportion	A.35 B.37	No
5	1	Yes (obstetrician, anesthetist)	UK	No	No	EL SC, Maternal indication, Severe kyphoscoliosis and limited neck motility	38	Unsuccessful epidural, general anesthesia adapted intubation for expected Grade II into clinical grade III Airway
6	1	Yes, internal medicine, anaesthesiologist	UK	UK	UK	EL SC maternal indication kyphoscoliosis and respiratory compromise	38	Spontaneous active in labour 1 week before elective SC. Continuous spinal anesthesia
7	1	UK	No	Yes. US at 34 wks: abnormal position hands and feet, low set ears and hairline	No	EL SC on maternal indication, kyphoscoliosis	37	Uneventful Regional/ general anaesthesia not known
8	1	No	UK	UK	Anesthetic consultation 1 day prior to elective SC. SC was postponed 2 days to gather info	EL SC	38	Uneventful. Combined spinal epidural anesthesia, walking after 1 day
9	1	Yes: obstetrics, internal medicine, anesthetics thromboprophylaxis 1st trimester -6 wks postpartum, surveillance lung capacity	No, was declined	No, US at 16 weeks	Hospital admission for breathlessness at 16 and 29 weeks	EL SC maternal indication breathlessness	30	No. ‘Awake’ fibre optic intubation required, general anaesthesia
10	1	UK	Amniocentesis and screening for viral infections unmarkable	Yes: contractures feet, hands, polyhydramnios, reduced rolling movements, small gastric bladder	No	EM SC, Fetal: Abnormal CTG and meconium stained fluid	38	No, General/ regional anesthetics not known
11	1	Yes (anesthetic, neonatal, obstetric and midwifery staff, clinical genetics)	Waived by the parents	No, ultrasound serial from 24 wks onwards	30 wk breathlessness Corticosteroids	EL SC, maternal indication breathlessness No thromboprophylaxis	31	No, general anesthesia
12	1	UK	UK	No, US at 36 weeks	No	Spontaneous labour, EM SC for cephalopelvenic dysproportion, narrow pelvic inlet	36	No, general anaesthesia
13	1	No	After pregnancy, daughter TPM2 mutation p.R133	Yes, US clenched hands, talipes equinovarus	No	EL SC Maternal, cephalopelvine, narrow pelvis	38	No, unknown general / local anesthesia
14	1	Yes (obstetrician, anesthetist and neonatologist)	No	No, serial US 1st, 2nd, 3rd trimester	No. Cervix length measurements and thromoboprofylaxis	EL SC maternal indication suspected cephalopelvine dysproportion, narrow pelvis and limited tight abduction. Vaginal delivery dissuaded for this reason	38	No, general anaesthesia
15	1	UK	UK	UK	Spontaneous preterm labor, Transverse lie fetus, fully dilated cervix	EM SC, maternal vocal cord surgery and failed epidural analgesia and the urgency of the situation	25	Epidural L4-5 insufficient for surgical anesthesia, mask induction and maintenance with nitrous oxide. Oxygen 50%, sevuflurane 1%, spontaneous breathing. No complications
16	1	Yes (gynaecologist and anesthesiologist)	UK	No	No	EM SC, Maternal indication, failed induction of labour, no fetal distress	37	Combined spinal and epidural L4-5, same as during labour
17	1	Yes (gynaecologist, anesthesiologist and cardiologist)	No	No, US at 22 wks	No	EL SC, maternal indication limited motility in hips. No thromboprofylaxis normal lung and heart function	38	No. Epidural
18	1	No	UK	Yes, US 16 wks: contracted fingers, 30 min no finger extension, US 20 weeks no finger movements	No	EM SC, maternal indication, hip dislocations	36	No. General/ regional anesthesia not mentioned
19	1	UK	UK	Yes	No fetal movements during pregnancy, only “flitters”	Spontaneous immature twin delivery	20	-
20	1	UK	UK	Yes, clenched hands with overlapping fingers	UK	UK	40	UK
21	1	UK	UK	UK	UK	UK	UK	UK
22	1	UK	No	UK	Oligohydramnios, decreased fetal movements during pregnancy	Vaginal	41	UK
23	2	UK	UK	UK	UK	UK	UK	UK
24	1	Yes, also anesthesiologist	UK	UK	UK	EL SC	38/40	Spinal anesthesia after a MRI lumbar spine to exclude neurological abnormalities
25	2	UK	UK	UK	UK	Vaginal EL SC due to breech position	2 × term	UK
26	1	UK	No	Yes, flexed wrists and clubfeet	Hypertension	EL SC due to maternal indication (preeclampsia)	38	UK
27	1	UK	UK	UK	UK	UK	UK	UK
28	1	UK	UK	UK	UK	UK	UK	UK
29	1	UK	Yes	Yes	UK	Termination of pregnancy due to US abnormalities (clubfeet)	UK	-
30	5	UK	UK	No	UK	UK	UK	UK
31	2	UK	UK	UK	UK	UK	UK	UK
32	UK	UK	UK	UK	UK	UK	UK	UK
33	1	UK	UK	UK	UK	UK	UK	UK
34	1	UK	UK	UK	UK	UK	UK	UK
35–43	40	UK	UK	UK	UK	UK	UK	UK

The obstetric outcomes of cases 1–26 were categorized by group. In Group 1 (n = 7), one woman (14%) had a vacuum-assisted vaginal delivery, three (43%) underwent cesarean sections, and the mode of delivery was unknown for the remaining three (43%). In Group 2 (n = 16), four women (25%) had vaginal deliveries, 11 (69%) underwent cesarean sections, and one experienced a preterm labour at 22 weeks. In Group 3 (n = 2), both women had cesarean sections.

AMC was suspected prenatally in 9 of the 82 pregnancies (11%), with contractures in hands and feet in 5, only in the hands in 3, and only clubfeet in 1 (Table 2). The 43 women gave birth to at least 71 liveborn children, with two neonatal deaths as a result of AMC in one case and in another case due to cardiorespiratory failure in a newborn with osteogenesis imperfecta and fractures. Postnatally, AMC was diagnosed after birth in 49% of the liveborn children (35 of the 71). Neonatal feeding problems or a pharyngeal obstruction were reported in cases 7, 10 and 13. Admission to a Neonatal Intensive Care Unit (NICU) was mentioned once, due to respiratory distress after a labour at 38 weeks. All neonatal outcomes are presented in Table 3.

**Table 3:** Postnatal outcomes, including maternal and neonatal complications

Case	Difficulties (maternal)	Difficulties (neonatal)	Child with AMC? (number/total children)	Birth weight (gram, percentile)
1	No	No	1/1	3700, 75th
2	No	No Died on day 5 of another autosomal dominant disease osteogenesis imperfecta, born with fractures and unexplained cardiorespiratory failure No	3/3 3 × affected hands, feet, facial anomalies	3100, 10th 2700, 3rd 3100, 10th
3	No	No	0/1	2740, 14th
4	No	No	2/2 fingers, hips, feet wrists, hands, feet and also limited knee movements and micrognathia	2120, 25th 2180, 10th
5	No	No	0/1	Appropriate growth for gestational age
6	No	No	0/1	UK
7	No	feeding difficulties, gastrostomy tube, 18 month	1/1elbows, wrist, fingers, hips, knees, feet facial, limited mouth opening, Identical to the mother	2400, 25th
8	No, discharged 3 days postoperatively	No	0/1	UK, healthy
9	No, walked with crutches within 2 days after operation. Thromboprofylaxis for 6 weeks postpartum	No, thrived well, home after 6 weeks	0/1	1300(girl), 10-50th
10	No	Intubation of the fetus due to functional pharyngeal obstruction, followed by tracheostomy	1/1, feet, hands, polyhydramnios, reduced rolling movements, small gastric bladder Functional pharyngeal obstruction caused by the Freeman-Sheldon Syndrome	3420, 50-90th
11	No	No, thrived well	0/1	1100, < 3rd
12	No	No	0/1	2500 gr, 10-50th
13	No	Feeding problems due to micrognathia. 10 days after birth 1 week hospitalized because of aspiration pneumonia, 4 weeks after birth resuscitation because breathing difficulties 1 h after bottle feeding, not successful, child died. Suspected aspiration pneumonia	1/1, Hands, feet, facial abnormalities, triangular face, downslanting palpebrae fissures, small mouth	2560, 3rd-10th
14	No	No, follow-up through 6 months	0/1	2755, 5-10th
15	No, postpartum + 6 days home	No	UK	UK
16	No, postpartum normal	No, stable	0/1	UK
17	No, after 3 days to home	No	0/1	3020, 10-50th
18	No	No	1/1, Distal arthrogryposis, arachnodactyly, and hip dislocation	2470, 10-50th
19	No	2 × Death caused by immaturity	2/2 Contractures feet, knees, hips, hands, mild scoliosis, retrognathia, pterygium colli	340 and 420 (twin)
20	UK	No	1/1	3124, 10-25th
21	UK	UK	1/1	UK
22	UK	No	1/1 upper and lower limbs	2430, < 3rd
23	UK	UK	0/2	UK
24	UK	UK	UK	UK
25	UK	UK	1/1	3700, 62nd 3120, 17th
26	No	NICU admission due to respiratory distress	1/1	2460, 5th
27	UK	UK	1/1, hand	UK
28	UK	UK	1/1, hand	UK
29	UK	-	1/1, hand	UK, termination of pregnancy
30	UK	No	0/5	UK
31	UK	UK	UK	UK
32	UK	UK	UK	UK
33	UK	UK	1/1 Clinodactyly and camptodactyly	UK
34	UK	UK	1/1 Upper and lower limbs, fingers, feet, hips	3600, > 10
25–43	UK	UK	16/31	UK

### AMC stability during and after pregnancy

Lung problems were mentioned in three manuscripts on three women, of whom two were grouped into Group 2 and one in Group 3. In one case (case 9) with a severe kyphoscoliosis, admission at 16 weeks gestational age was reported due to this problem. The uterine fundus was at xyphoid level. Antenatal corticosteroids were administered due to breathlessness and dosage of inhaled agonist and steroid was increased along with chest physiotherapy and upright position during sleep enabled continuation of the pregnancy till a gestational age of 29 weeks. Case 11 also reported breathlessness during pregnancy. Another woman (case 6) underwent an elective section cesarean due to a kyphoscoliosis and respiratory compromise. From these 3 women with maternal difficulties, none had a child affected by AMC. No reports were found in the included manuscripts on fatigue during or after pregnancy, hyperemesis, gestational diabetes, use of medication such as pain reliever, or anemia. Comments on challenges caused by maternal AMC during and after pregnancy are presented in Tables 2, 3, respectively.

### Counselling aspects in AMC and pregnancy

The utilization of pre-pregnancy counselling, a medical consultation before pregnancy aiming to optime health and to address potential pregnancy-related risks, was described in one manuscript [37]. Counselling advice to perform before, during and after pregnancy from the included manuscripts are grouped in Table 4.

**Table 4:** Overview of advice mentioned in the included articles from the literature search AMC and pregnancy

Period	Counselling aspect	Explanation
Prepregnancy	Genetic counseling	Geneticists informs about the types of AMC and update on possible genetic tests [1]
	Prepregnancy counselling	Multidisciplinary approach [8, 35] Contraceptive advice [35] Discussing facts and challenges of pregnancy and AMC [37] Respiratory function test if applicable [37]
Pregnancy	Medical history	Obstetric history (prior pregnancies, mode and time of delivery, birth weight) [24, 25] Prior operations, including type of anesthesia and possible advices [24, 27] Family history [30] Use of medication [30] Mobility: independent walking, walking with aids, wheelchair bound, immobility [27]
	Physical examination	Physical examination with extra focus of members of multidisciplinary team (e.g. gynaecologist, internist, pulmonologist, anesthesiologist) [25, 27] Weight, height, BMI [25] Extremities including mobility (including range of movements of the joints) [8] Cardiovascular and respiratory system [40] Head and neck area (e.g. micrognathia or high arched palate), including Mallampati score and neck mobility [24, 25, 39] Shoulders (e.g. deformity of the scapula) [25] Spine (e.g. scoliosis, spina bifida, sacral agenesia or vertebral anomalies) [25] Cardiovascular system (e.g. heart diseases) [25] Respiratory system (e.g. tracheoesophageal fistula or hypoplastic lungs) [25] Genitourinary system (e.g. rectal or labial defects) [25] Abdomen (e.g. inguinal hernia) [25] Venous access evaluation [30]
	Home management	Needs for home management dependent of mobility (aids) [8] Local occupational therapist (for home modifications) [8] Social worker [8]
	Tromboprophylaxis	Tailored counselling about using thromboprophylaxis during and after the pregnancy [28, 31]
	Cervix length measurements	Suggested in relation to increased risk of preterm labour [34]
	In case of breathlessness (AMC related)	Monitoring cardio-respiratory condition [31] Peak expiratory flow rates measurements [31, 37] Chest radiography (signs of infection?) [34] Evaluation of the uterine fundus in relation to diaphragm [34] Steroids for maternal lungs [34] Chest physiotherapy^.^ [34] Advice upright sleeping position to reduce elevation of the diaphragm [34] In case of worsening: counselling about continuation or terminating the pregnancy [34]
	Serial ultrasound investigations	Healthcare providers should be aware of recurrent AMC in the fetus [8, 37]: - Features due to limited motility: joint contractures (e.g. clubfoot), micrognathia, decreased fetal movements, altered amniotic fluid - Associated anomalies: brain and hearth anomalies, heart, joint webbing - Fetal growth restriction
	Prenatal testing	First trimester test for aneuploidies [37] Genetic counselling about prenatal invasive testing: risk calculation for fetal AMC [24, 37] Genetic testing update possibilities(chromosomal or monogenic) [31]
	Anesthetic	
	Anesthetic assessment	Early in pregnancy anesthetic assessment [30, 39] Expected difficulties during administration of analgesia [30, 39] Craniofacial evaluation: cleft palate, laryngeal stenosis, craniosynosthosis, micrognathia Spinal abnormalities: scoliosis, spina bifida, or sacral agenesis could have abnormal cerebrospinal fluid dynamics [25, 39] Expected anesthetic problems during infusion placement (e.g. due to joint contractures or scarring), or insertion the catheter of the regional analgesia (e.g. due to spinal anomalies) [25] Choice of anesthesia technique should be tailored to the individual patient's anatomy, overall health, and the specific surgical procedure to optimize safety and efficacy [25]
	General versus regional analgesia	Weigh the potential difficulties and risks [27, 31, 34]: - Additional risks during general analgesia compared to regional analgesia are: difficulties during intubation due to a limited neck mobility and problems related to the decreased cardiopulmonary function - Patients with AMC could react unpredictable on medications (e.g. muscle relaxants and inhalation anesthetics). Therefore, proper dosing and careful monitoring are crucial [25, 37] - Spinal analgesia could be challenging in patients with AMC who have spine deformities (e.g. scoliosis) Therefore, identifying and targeting nerves for blocks may be more difficult due to the altered anatomy
Delivery	Mode of delivery	Counselling about the mode of delivery, individualized and dependent of maternal and fetal investigations [8]
	Timing of delivery	In general term age. Challenge in case of for example maternal pulmonary discomfort (e.g. breathlessness) before term age, while the fetus is a good condition [31] A pulmonary function test is suggested after 28 weeks gestational age in symptomatic patients and an electrocardiogram in asymptomatic patients and adjustments in more upright sleeping position [34]
Postpartum	Maternal	
	Home management	Modifications to a bassinet to enable self-sufficient care of the newborn [8, 34] Social service provision: need for carers and housing [34]
	Thromboprophylaxis	Continuation of 6 weeks, in line with recommendation of the Royal College of Obstetricians and Gynaecologists [31]
	Physical examination (neonatal)	Joint stature including range of motion, features of AMC, general physical evaluation, and birth weight [24]

## Discussion

This study makes a significant contribution in filling the knowledge gap concerning pregnancy-related topics in 43 women with AMC. The outcomes of pregnancy were reviewed in 82 pregnancies published during a 40-year period from 1984 to 2024. This information can serve as an important support for healthcare professionals who provide care for women with AMC and for the AMC community.

### Maternal, fetal and neonatal outcomes

The characteristics of the women with AMC affect an about equal distribution of the AMC Groups 1 and 2. Notably, only two women were diagnosed with Amyoplasia, which is in contrast with the typical distribution observed in live-born children with AMC, where about a third has Amyoplasia [1]. This suggests that the review may not fully represent the general population of individuals with AMC. The low occurrence of AMC Group 3 with musculoskeletal involvement plus central nervous system dysfunction, namely 2 of the 43 women (5%), might be attributed to the severity of these conditions. For example, these individuals are typically more severely impacted, and may therefore have lower pregnancy rates. Regarding physical ability, at least 26 women (60%) achieved mobility independence, aligning with the 52% observed in a cohort of 177 individuals with AMC [9].

Details on pregnancy-related outcomes could be depicted from 26 of the 43 women (cases 1–26) with in total 31 pregnancies. The mode of delivery among these women was in one-quarter a vaginal delivery (8 of the 31 pregnancies) and in the remaining 74% a cesarean section. The percentage of cesarean sections among cases 1–26 was higher than in the general American population (30–32%) [47]. Among this group, the distribution of elective and emergency cesarean section was 17 (74%) and 6 (26%), respectively. The main reason for an elective cesareans were suspected cephalopelvic disproportion and for the emergency cesarean section lack of progress during labour. Breathlessness caused by the AMC and the expanding gravid uterus was the reason to perform an elective cesarean section three times (cases 6, 9 and 11). The reported percentage of preterm deliveries (< 37th week) among cases 1–26 was 22% (7 of the 31), with a median at 31 weeks (range 20–36 weeks). This finding is also higher than in the worldwide general population observed 12% [48]. One-quarter (6 of the 24) of all infants with a reported birth weight had a birth weight below the 10th percentile. A recent study confirmed a smaller weight in 206 American children with AMC in comparison to typically developing children during the first 36 months of life [49]. A higher maternal risk of adverse outcomes was also observed in a recent retrospective study among 2074 women with a physical, intellectual, and sensory disability [16]. This study showed higher rates of cesarean sections and premature rupture of membranes in women with a disability compared to those without [16].

These 43 women gave birth to at least 71 liveborn children, with two neonatal deaths as a result of AMC in one case and in another case due to the coexisting osteogenesis imperfecta (cases 2 and 13). One woman had a spontaneous and immature delivery of twins affected by AMC at 20 weeks (case 9). Another woman with Sheldon Hall syndrome terminated the pregnancy due to a pathogenic variant in the TNNT3 gene (case 29). In this case, prenatal ultrasound examinations had shown abnormalities and prenatal invasive testing confirmed that the fetus was also affected. Additionally, prenatal invasive testing was reported in another case, but the results were not discussed and the author did not mention which genetic tests were performed (case 10).

Sonographic structural examination led nine times to a prenatal suspicion of fetal AMC. Additional descriptive fetal motor assessment was described in two manuscripts [33, 41]. Maternal perceived fetal movements were mentioned in six cases (1, 3, 10, 18, 19 and 22) and serial ultrasound investigations were performed in six cases (1, 2, 4, 11, 14 and 18). Serial examinations are the advised manner to observe if the phenotypical features of AMC worsen over time [50–52]. Finally, AMC was diagnosed after birth in 49% of the liveborn children (35 of the 71). This high percentage can be explained by the high percentage of autosomal dominant inheritance in this population.

Over time, new genetic techniques have been developed. While there are nine women with a proven genetic abnormality in the current study, we assume that more women had a genetic disorder who have never been tested. In this population, the inheritance of 14 of the 43 mothers was not known. A genetic diagnosis could help to confirm the genotype of AMC and to estimate the recurrence rate [53]. Recently, Laquierriere et al. emphasized the additional value of Whole Exome Sequencing to targeted exome sequencing in a population of unrelated parents from children with AMC [54]. Therefore, a close collaboration is crucial between clinical geneticist and obstetrician who should be up to date on new genetic testing possibilities.

### AMC stability during and after pregnancy

Stability of AMC during and after pregnancy did not deteriorate in most of the included cases, as far as this was described in the included manuscripts. There were three exceptions. Three women experienced breathlessness during pregnancy (cases 6, 9 and 11). In all cases it was caused by the combination of small stature and scoliosis. Only one other manuscript described immobility and pain [8]. The latter is unexpected since pain is commonly experienced in adult populations with AMC and also in pregnant women without AMC [9, 55, 56].

### Counselling aspects in AMC and pregnancy

A few aspects for (pre) pregnancy counselling for women with AMC will be highlighted. Firstly, the importance of a multidisciplinary approach was emphasized by various authors [8, 25, 27, 31, 34, 37, 39, 40, 44]. The team should be tailored to the type of AMC (e.g. gynecologist, neurologist, anesthesiologist, rehabilitation doctor, neonatologist, physiotherapist and/or social worker). Secondly, while a pre-pregnancy counselling was described in only one manuscript, we emphasize its importance [37]. A pre-pregnancy counselling should focus on the understanding of the impact of AMC on pregnancy and vice versa. This stepwise approach evaluating the opportunities and challenges is similar to individuals with other relatively rare chronic diseases like systemic lupus erythematosus and kidney disease [57–59]. Providing women with AMC a (pre)pregnancy counselling is advantageous in preparing them for potential challenges during pregnancy, for example the respiratory system’s impairment leading to maternal discomforts such as breathlessness, potential anesthetic difficulties and increased risk of thromboembolisms caused by decreased mobility [37]. Closer follow-up and pre-pregnancy counseling are recommended, particularly for women with AMC who have short stature and scoliosis, as they appear to experience more complications. Additionally, factors such as difficulties with intravenous access due to prior frequent venipunctures, thin subcutaneous tissue, and challenges in positioning for procedures should be considered in their clinical management. In addition, it is advisable to have medical follow-up examinations for pregnant women with AMC in a secondary or tertiary healthcare center, according to existing comorbidities. The accessibility of the airway should always be checked in patients with AMC since limitations have been reported in 25% of these patients [60]. Regional anesthesia could be advantageous in these cases, but it could also be challenging in case of a scoliosis [60, 61]. A difficult intubation was described in two of the included manuscripts [25, 38]. The total number of cases with a general anesthesia is unknown. In case of severe airway obstruction, resorting to a tracheostomy may be a final option in patients with AMC [60, 61]. Nothing related to optimize intraoperative position or intravenous access was reported in any of the cases, despite the significance of these aspects [60].

### Strengths and limitations

The strength of our literature search lies within the systematic approach of evaluating case reports concerning women with AMC with a pregnancy. The obtained knowledge facilitates information and advice in detail for professional healthcare providers and women with AMC. Most information on this rare disorder has been obtained concerning women with AMC group 1 and 2, especially Distal arthrogryposis of various types (1 and higher) and limited to Amyoplasia. A limitation is that despite the extensive  literature search spanning four decades (1984–2024), the number of included cases is still modest. Moreover, not all manuscripts have been set-up with the goal in mind to examine the influence of AMC on pregnancy and vice versa. Therefore, no details on obstetrical outcome parameters could be given in a considerable proportion. We are aware of the Bamshad classification on arthrogryposis making a precise distinction between various forms of distal arthrogryposis based on neurological examination and genetic findings [62]. The manuscripts of our study examined a period lacking this detailed information. On the other hand, all present individual data of the included women with AMC and their pregnancy outcome are listed systematically.

### Future research

Future research with a larger sample size should strive to register prospectively influence of AMC on pregnancy and vice versa in women with different types of AMC. With this purpose in mind, a minimal common data set for an AMC register, inclusive pregnancy outcome has been designed by means of a multidisciplinary Delphi procedure inclusive patients with AMC [63]. Furthermore, more information is needed on aspects during delivery (e.g. leg positioning during vaginal or operative labour, pain relief during a vaginal labour) and postpartum period (e.g. breastfeeding instructions with the affected limbs).

## Conclusion

 This scoping review is an initial step in addressing the knowledge gap on the obstetrical outcome in women with AMC. The findings of this review underscore the importance of (pre-)pregnancy counselling concerning the mode of delivery, possibility of preterm birth, and stability of AMC (worsening of symptoms due to contractures, increased muscle weakness, pain or lung involvement). The relevance of the obtained information regarding possibilities and challenges is particularly strong for women with Distal Arthrogryposis and may not be directly applicable to other types of AMC. Further prospective studies are needed to provide more information in a populations with a wider spectrum of AMC, especially Amyoplasia. The wide spectrum of the AMC phenotypic expression and underlying causes requires a multidisciplinary tailored approach to reduce the risks of cesarean section, preterm labor, and having a small-for-gestational-age child. Additionally, it is crucial to address respiratory difficulties in cases of scoliosis and potential challenges during anesthetic procedures.

## Supplementary Information

Additional file 1

Additional file 2

## Data Availability

Available on request.

## Abbreviations

AMC: Arthrogryposis multiplex congenita
FADS: Fetal akinesia deformation sequence
M.D.: Doctor of Medicine
Ph.D.: Doctor of Philosophy
UMC: University Medical Centers
