# Supplementary material for: Maternal, fetal and neonatal outcomes among pregnant women with arthrogryposis multiplex congenita: a scoping review
Source: Orphanet J Rare Dis. 2025 Mar 17;20:129. doi: 10.1186/s13023-025-03631-5 (PMC11912775; doi:10.1186/s13023-025-03631-5)
Supplement: Supplementary file 1 — Additional file 1 [file 13023_2025_3631_MOESM1_ESM.docx]

**Additional File Table 1 Query developed to search literature for pregnancy related topics in women with AMC, December 2021 and August 2024**

| **Query** |
| --- |
| ("Arthrogryposis"[MeSH Terms] OR "arthrogrypos*"[Title/Abstract] OR "congenital arthromyodysplasia*"[Title/Abstract] OR "guerin stern syndrome*"[Title/Abstract] OR "Myodystrophia Fetalis Deformans"[Title/Abstract] OR "arthrogryposis multiplex congenita*"[Title/Abstract] OR "congenital multiple arthrogrypos*"[Title/Abstract] OR "rocher sheldon syndrome*"[Title/Abstract] OR "rossi syndrome*"[Title/Abstract] OR "amyoplasia congenita*"[Title/Abstract] OR ("Dystonia Musculorum Deformans"[MeSH Terms] OR "Dystonia Deformans"[Title/Abstract] OR "torsion dystonia*"[Title/Abstract] OR "idiopathic torsion dystonia*"[Title/Abstract])) AND ("Pregnancy"[MeSH Terms] OR "Pregnant Women"[MeSH Terms] OR "Preconception Care"[MeSH Terms] OR "Prenatal Education"[MeSH Terms] OR "pregnan*"[Title/Abstract] OR "gravidit*"[Title/Abstract] OR "gestation*"[Title/Abstract] OR "placentat*"[Title/Abstract] OR "prepregnan*"[Title/Abstract] OR "conception*"[Title/Abstract] OR "preconception*"[Title/Abstract] OR "prenatal*"[Title/Abstract] OR "parent*"[Title/Abstract] OR "childbirth*"[All Fields] OR "birth*"[Title/Abstract] OR "deliver*"[Title/Abstract] OR ("Surveys and Questionnaires"[MeSH Terms] OR "questionnaire*"[Title/Abstract] OR "instrument*"[Title/Abstract] OR "measure*"[Title/Abstract] OR "survey*"[Title/Abstract])) |
